# Opinions of South African optometry students about working in rural areas after graduation

**DOI:** 10.4102/phcfm.v7i1.799

**Published:** 2015-07-31

**Authors:** Khathutshelo P. Mashige, Olalekan A. Oduntan, Rekha Hansraj

**Affiliations:** 1 Discipline of Optometry, School of Health Sciences, University of KwaZulu-Natal, South Africa

## Abstract

**Background:**

Eye and vision problems have been reported to be more prevalent in rural than urban areas; and a large proportion of South Africans live in the rural areas.

**Aim:**

To investigate the opinions of South African optometry students about working in rural areas after completion of their training and to identify factors that may influence their decisions.

**Method:**

This was a cross-sectional quantitative study using a survey instrument containing both closed and open-ended, semi-structured questions.

**Results:**

Four hundred and thirty-eight students responded to the questionnaire (85.4% response rate). Overall, many of the respondents did not want to open their first (66%) or second practices (64.6%) in the rural areas. However, most respondents from rural backgrounds reported that they would open their first (77.2%) or second (79.4%) practice in the rural areas. The main reasons cited by the respondents for their unwillingness to work in the rural areas were financial concerns (81.2%), personal safety (80.1%) and poor living conditions (75.3%), with a significantly higher number (*p* < 0.05) being from urban respondents for the latter two issues only.

**Conclusion:**

Many students were not in favour of opening practices in rural areas, but were willing to work for the government or a non-governmental organisation after graduation. Efforts should be made to address financial incentives, safety and living conditions in the rural areas. The results of this study have implications for the future of availability and accessibility of eye care services to those living in the rural and remote areas of the country.

**Opinions des étudiants sud-africains en optométrie sur la possibilité de travailler dans les zones rurales après l’obtention de leur diplôme.**

**Contexte:**

Les problèmes des yeux et de vision sont plus courants dans les zones rurales qu’en ville; et une forte proportion de Sud-africains vit dans les zones rurales.

**Objectif:**

Examiner les opinions des étudiants sud-africains en optométrie sur la possibilité de travailler dans les zones rurales après avoir terminé leur formation et identifier les facteurs pouvant influencer leur décision.

**Méthode:**

C’est une étude quantitative transversale utilisant un instrument de sondage contenant des questions semi-structurées fermée et ouvertes.

**Résultats:**

Quatre cent trente-huit étudiants ont répondu au questionnaire (un taux de réponse de 85.4%). En général, un grand nombre de répondants ne voulaient pas ouvrir leur premier (66%) ou deuxième cabinet (64.6%) dans les zones rurales. Cependant, la plupart des répondants originaires de la campagne ont répondu qu’ils ouvriraient leur premier cabinet (77.2%) ou leur second (79.4%) dans les zones rurales. Les raisons principales citées par les répondants pour ne pas vouloir travailler dans les zones rurales étaient des préoccupations financières (81.2%), la sécurité personnelle (80.1%) et les mauvaises conditions de vie (75.3%), avec un plus grand nombre (*p* < 0.05) de la part des répondants urbains pour les deux derniers problèmes.

**Conclusion:**

Beaucoup d’étudiants ne voulaient pas ouvrir de cabinet dans les zones rurales, mais étaient prêts à travailler pour le gouvernement ou une organisation non-gouvernementale après l’obtention de leur diplôme. Il faudra s’occuper des incitations financières, de la sécurité et des conditions de vie dans les zones rurales. Les résultats de cette étude ont des implications pour le futur de la disponibilité et de l’accessibilité des services de soins oculaires pour ceux qui vivent dans les zones rurales et les régions reculées du pays.

## Introduction

Visual impairment (low vision and blindness) is a major health problem worldwide, the prevalence being likely to increase significantly as the percentage of elderly people increases, as this section of the population is often affected.^1^ Whilst most cases of visual impairment are preventable or manageable by surgery and/or refractive error corrections,^2,3,4,5^ the prevalence rates remain high in many countries. This is particularly the case in developing nations, for a variety of reasons, including the following:

- **Availability**: Eye care services are not readily available, either because of a lack of trained personnel or because eye care practitioners are concentrated in urban areas.^6,7,8,9^ Consequently, people in rural areas with treatable eye conditions are largely unattended to, whilst city facilities remain underutilised.^10,11^
- **Accessibility**: Many people, particularly those in rural areas, do not have access to eye care services. Factors such as lack of funds for transport to eye care facilities may result in poor access to eye care services.^12,13^
- **Affordability**: As eye care services are often provided by private practitioners or on a cost-recovery basis, rather than being freely available through government facilities, many people cannot afford them.^14,15,16,17^

In view of the fact that many causes of visual impairment are preventable, the World Health Organization (WHO) and the International Agency for the Prevention of Blindness (IAPB), with an international membership of non-governmental organisations (NGOs), professional associations, eye care institutions and corporations, developed the global Vision 2020 initiative: the Right to Sight Campaign.^18^ This initiative has set goals to eliminate preventable blindness by the year 2020.^18^ According to the WHO in 2000,^18^ one of the most complex challenges facing policy makers in all countries is ensuring that people living in rural and remote areas have access to trained health workers. Worldwide, a shortage of qualified health workers in remote and rural areas impedes access to healthcare services for a significant percentage of the population, slows progress toward attainment of the Millennium Development Goals (MDGs) and challenges aspirations of achieving the WHO initiative, Health for All.^18^

As with many developing countries, poor access to eye care services is one of the major healthcare challenges facing South Africa.^19^ A large proportion of South Africans live in rural areas,^20^ where reports indicate poor access to eye care services. For 2014, Statistics South Africa estimated the mid-year South African population to be approximately 54 million, using the cohort-component methodology.^21^ Forty-two-and-a-half per cent of the South African population lived in rural areas, according to the latest available data.^22^ A recent study by Thivhafuni^23^ found poor availability, accessibility and affordability of optometric eye care services in the Mutale Municipality of Vhembe District, Limpopo Province, South Africa. As with many other rural areas, this is because most optometric services in the country are privately owned, making them unaffordable to many citizens, specifically those living in rural areas.^19,24^

No studies were found that explored the reasons for poor eye care services in rural areas, with a number of authors^25,26^ having speculated about having access to a sustainable market who can afford their services. This article therefore aims to investigate South African optometry students’ opinions about working in the rural areas after graduation, along with factors that may influence such views. Some of the recommendations of the WHO^27^ for improving access to healthcare in rural and remote areas will be examined in this study.

## Research methods and design

### Design

This study followed a quantitative cross-sectional research design in the form of a questionnaire survey of all of the 2013 South African optometry undergraduate students. All the undergraduate optometry students (first year to fourth year) who agreed to participate in the study were included. The study was conducted at all four institutions offering optometry courses in the country: universities of Free State (UFS), Johannesburg (UJ), KwaZulu-Natal (UKZN) and Limpopo (UL). Each department offers a four-year Bachelor of Optometry (BOptom) degree.

### Questionnaire

The content and design of the questionnaire was based on a review of other studies,^20,28,29,30^ being modified to suit the objectives of the study. The survey took approximately 30 minutes to complete and consisted of 31 questions that explored the following areas:

1. Demographic characteristics: age, gender, marital status, race, institution of learning, year of study, province of origin and place of origin (rural versus urban).
2. Views about practice choice: to establish why they did or did not want to practise in rural areas. This was assessed with questions such as: ‘If you were to start a practice, would you establish your first practice in the rural area? – “YES or NO”?’.
3. Possible attractions and incentives: a list of factors was provided and the students indicated whether they were ‘very’, ‘moderately’, ‘slightly’, ‘not really’ or ‘not at all’ attractive with regard to the desire to engage in rural optometric practice.

### Procedure

All first- to fourth-year South African students were included in this study, with non-South Africans being excluded. A self-administered questionnaire written in English, the official language of the optometry training in South African institutions, was used. Copies of the questionnaire were distributed by hand between June and October 2013 to all optometry students who agreed to participate in the study by staff at each institution.

### Data analysis

Data were captured and analysed with the Statistical Programme of Social Sciences (SPSS), version 19.0 (IBM SPSS Inc., Chicago, IL 2010). Responses from the open-ended questions were grouped into common themes and collated. The number of respondents stating a particular response was noted. Descriptive statistics were used to establish values such as means, standard deviation, frequencies and percentages of the collected data. Chi-square tests were used to establish associations between relevant variables. A *p*-value of < 0.05 was considered statistically significant.

### Ethical considerations

Ethical approval to conduct the study was obtained from the Human and Social Sciences Research and Ethics Committee of the University of KwaZulu-Natal (Reference Number: HSS/0067/013). Permission to collect data was obtained from the Head of each Optometry Department at all four universities that offer the programme in South Africa. All those who took part in the study signed the consent form and were provided with the necessary information.

### Trustworthiness

The data were collected by an optometric staff member in each of the four institutions. The sample size was high enough to represent the study population. Participants were encouraged to be frank and honest in their responses, with the assurance that their identity will be kept confidential. All literature sources, data and materials utilised and represented by the authors in the paper are credible, dependable and can be verified.

### Reliability

Prior to the main study, a pilot study was conducted using 10 students who were not part of the main study to establish the appropriateness of the study procedures, the methods and logistics. These participants were given the questionnaire for completion and retested after four weeks. The responses obtained during the two sessions were similar. All queries that arose from the pilot study were addressed and the questionnaire modified accordingly before the main study was conducted.

### Validity

The validity of the findings in this study was ensured by using an adequate sample size and by including all the optometry departments in South African universities. Optometric staff members in each institution distributed and collected the questionnaire, after which a qualified statistician provided expert advice and support with the data analysis.

## Results

The results are presented with respect to the three categories of the questionnaire: demographic characteristics; their views about working in the rural areas; and possible attractions and incentives.

### Demographic characteristics

Of the 513 eligible South African undergraduate optometry students who were registered in 2013, 438 completed the questionnaire, giving a response rate of 85.4%. Their ages ranged from 17 to 35 years, with a mean of 21.1 ± 1.8 years. The majority (*n* = 294; 67.1%) were women, had never married (97%), and were from urban areas (*n* = 285; 65.1%). Slightly less than one-quarter (*n* = 145; 23.3%) of the respondents were from the UKZN, and 133 (30.4%) were in their first year of optometric study (Table 1). The majority of optometry students (*n* = 123; 98%) at University of Limpopo (UL) were black people, whilst there were more white students at the UFS (*n* = 64; 85.7%) and UJ (*n* = 57; 61.1%), respectively. At UKZN, 75 (52%) of the students were black people and 54 (37%) were Indians.

**TABLE 1:** Demographics of the 2013 South African optometry students (*n* = 438).

Characteristics	*n*	%
**Age**		
17–19	208	47.5
20–21	149	34
> 21	81	18.5
**Gender**		
Male	144	32.9
Female	294	67.1
**Race**		
Black people	173	39.5
Mixed-race	2	0.5
Indian	118	26.9
White people	145	33.1
**Marital status**		
Single	425	97
Married	13	3
**Place of origin**		
Urban	285	65.1
Rural	153	34.9
**Province of study**		
Eastern Cape	32	7.3
Free State	74	16.9
Gauteng	87	19.9
KwaZulu-Natal	102	23.3
Limpopo	96	21.9
North-West	16	3.7
Mpumalanga	13	3.0
Northern Cape	7	1.6
Western Cape	11	2.5
**Training institution**		
UFS	75	17.1
UJ	93	21.2
UKZN	145	33.1
UL	125	28.5
**Year of study**		
1st	133	30.4
2nd	118	26.9
3rd	97	22.1
4th	90	20.5

UFS, University of Free State; UJ, University of Johannesburg; UKZN, University of KwaZulu-Natal; UL, University of Limpopo.

Of the respondents from rural areas (*n* = 153; 34.9%), the majority attended primary (*n* = 138; 90.2%) and high schools (*n* = 125; 81.7%) in the same rural area. Most respondents from rural areas returned home for their holidays (*n* = 126; 82.4%) to visit family members (*n* = 148; 96.7%).

### Respondents’ views about working in rural areas

Overall, most respondents reported no desire to establish their first (*n* = 289; 66%) or second (*n* = 283; 64.6%) practice in a rural area. However, most respondents from rural backgrounds reported that they would open their first (*n* = 115; 77.2%), or second (*n* = 123; 79.4%) practice in a rural area. There was a significant difference between urban and rural respondents (*p <* 0.05) regarding practice location. Many respondents indicated that they would accept an offer from the government (*n* = 297; 67.8%) or an NGO (*n* = 280; 63.9%) to work in a rural area, with no significant difference between urban and rural respondents (*p* > 0.05). Of those who did not intend to work in rural areas, most cited finances (*n* = 356; 81.2%) and personal safety (*n* = 351; 80.1%) as being major concerns.

Although there was a significantly higher number (*p* < 0.05) of respondents from urban areas who reported not wanting to work in rural areas, there was no significant difference (*p* > 0.05) between urban and rural respondents regarding ‘financial concerns’ as an impediment to working in the rural areas. Sixty-one per cent indicated that a good salary would be a motivation for them to accept a government job offer in the rural area, there being no significant difference (*p* > 0.05) between urban and rural respondents. Most respondents (*n* = 305; 69.6%) did not agree with the proposed compulsory community service for graduating optometrists, with a significantly higher number being from urban respondents (*p* < 0.05) (Table 2).

**TABLE 2:** Respondents’ views about working in rural areas.

Variables	Characteristics	Rural		Urban		Total	
		***n***	**%**	***n***	**%**	***n***	**%**
Establish first practice in the rural area	Yes	115	77.2	34	22.8	149	34.0
	No	53	18.3	236	81.7	289	66.0
Establish second practice in the rural area	Yes	123	79.4	32	20.6	155	35.4
	No	79	27.9	204	72.1	283	64.6
Employed by government	Yes	153	51.5	144	48.5	297	67.8
	No	63	44.7	78	55.3	141	32.2
Employed by non-government organisation	Yes	153	54.6	127	45.4	280	63.9
	No	67	42.4	91	57.6	158	36.1
Reasons not to practise in the rural area	Financial concerns	150	42.1	206	57.9	356	81.3
	Personal safety	122	34.8	229	65.2	351	80.1
	Poor living conditions	99	30	231	70	330	75.3
	Language barrier	74	36.5	129	63.5	203	46.3
Motivation to accept government job in the rural area	Good salary	124	46.4	143	53.6	267	61.0
	Proximity to home	101	100	0	0	101	23.1
	Inability to find a job	33	36.3	58	63.7	91	21.0
	Help the community	113	80.7	27	19.3	140	32.0
Community service for graduates	Yes	82	61.7	51	38.3	133	30.4
	No	39	12.8	266	87.2	305	69.6

Regarding possible attractions and incentives, the students rated ‘financial incentives’ (*n* = 330; 75.3%), ‘acceptable working conditions’ (*n* = 325; 74.2%), ‘scholarship and bursary to study’ (*n* = 302; 68.9%) and ‘career ladder for rural health workers’ (*n* = 270; 61.6%) as very attractive factors that would influence them to work in a rural area (Table 3). A combination of ‘Moderate’ and ‘Very’ scores however, indicated that many students would be willing to work in rural areas.

**TABLE 3:** Factors that would influence their decision to engage in rural optometric practice.

**Factors**	**Level of attractiveness**					
	**Not at all *n* (%)**	**Not really *n* (%)**	**Slight *n* (%)**	**Moderate *n* (%)**	**Very *n* (%)**	**Moderate and Very combined**
Enhanced profiles of rural health workers	7 (1.6)	27 (6.2)	118 (26.9)	88 (20.1)	198 (45.2)	286 (65.3)
Outreach interaction between the rural and urban health workers	9 (2.1)	14 (3.2)	99 (22.6)	111 (25.3)	205 (46.8)	316 (72.1)
Facilities for knowledge exchange to reduce sense of professional isolation	5 (1.1)	32 (7.3)	81 (18.5)	90 (20.5)	230 (52.5)	320 (73)
Enhance scope of practice	7 (1.6)	20 (4.6)	79 (18)	116 (26.5)	216 (49.3)	332 (75.8)
Facilities for Professional Development	13 (3.0)	19 (4.3)	66 (15.1)	165 (37.7)	175 (39.9)	340 (77.6)
Scholarship and bursary to study	21 (4.8)	27 (6.2)	24 (5.5)	65 (14.8)	302 (68.9)	367 (83.7)
Career ladder for rural health workers	9 (2.1)	17 (3.9)	45 (10.3)	97 (22.1)	270 (61.6)	367 (83.7)
Better living conditions	13 (3.0)	13 (3.0)	32 (7.3)	62 (14.2)	318 (72.6)	380 (86.8)
Financial incentives	8 (1.8)	10 (2.3)	31 (7.1)	59 (13.5)	330 (75.3)	389 (88.8)
Conducive or favourable working environment	3 (0.7)	8 (1.8)	35 (8.0)	67 (15.3)	325 (74.2)	392 (89.5

## Discussion

Most students studying optometry were relatively young, possibly because the majority apply for admission to the BOptom programme during their matric (final) year. The gender distribution of the respondents indicated that the number of women was similar across all training institutions, with more women (67.1%) than men (32.9%) either seeing optometry as a career option or being accepted into the programme. These findings are similar to those of Mashige and Oduntan^24^ in 2011, who reported more female (69.5%) than male (30.5%) students study Optometry in South African institutions. Although the number of black students was on average higher than other race groups, historically white institutions still had the highest number of white students and a traditional black institution had the highest number of black students. Also noteworthy is the significantly low proportion of mixed-race students in all institutions. These racial disparities suggest that optometry departments at these universities should strengthen their efforts to fully effect a change in the student demographics. The majority of the students were from the provinces where institutions with optometry departments are located (Table 1). This again suggests the need for admission policies of training institutions to be re-evaluated and for there to be greater representation of students from provinces where there are no optometry departments. Mashige and Oduntan24 suggested that such steps could help to change the demographics and distribution of the optometric workforce in the country. The cohort of students surveyed in this study had strong urban representation and contributed a substantial proportion (65.1%) of respondents (Table 1).

In a developing country such as South Africa, rural community residents are at greater risk of having visual impairment because of a scarcity of eye care services. This scarcity is compounded by the poor distribution of the eye care practitioners who are concentrated in the urban areas, leaving the rural areas without services.^11,19,20^ In addition, non-availability,^6,7,8,9^ poor accessibility^12,13^ and non-affordability^14,15,16,17^ are barriers to eye care utilisation. These are the major reasons for a greater prevalence of visual impairment amongst residents of rural communities.

Our study also shows that the majority of students from a rural background were willing to return and work in their area of origin. Optometric institutions should attract students from rural areas with academic potential and, if necessary, provide them with academic support or extended curriculum programmes in order to ensure their success. Studies have suggested that graduates from rural areas are more likely to return to their home areas to practise,^31,32,33^ providing affordable eye care services and eliminating the costs of transportation to urban areas. It is important to emphasise that these four teaching institutions have no special admission criteria for rural students, their admission criteria being based on merit and not expected practice location. Efforts should therefore be made to ‘use targeted admission policies to enrol students with a rural background, in order to increase the likelihood of graduates choosing to practise in rural areas’, as recommended by the WHO.^27^ This is further supported in our study by the fact that whilst many respondents reported that they did not want to establish their first or second practice in a rural area, most respondents from rural backgrounds indicated that they would.

As with most health care facilities, the majority of optometric service facilities are located in the urban areas, leaving the majority of the citizens who live in the rural areas without such services. The South African government anticipates that the ‘certificate of need’ policy would contribute to reversal of this inequality in healthcare services, including optometric services. This policy means that a healthcare professional needs to get a certificate of need from the Director General of Health before establishing, constructing, modifying or acquiring a health establishment.^34^ Hence, optometrists who wish to open a private practice or branch(es) will have to apply to the Department of Health for this certificate to give them permission to work in their chosen area. In this study, the majority of respondents, irrespective of origin, indicated that they would gladly accept a government or an NGO offer to work in rural areas. This underscores the value and possible impact of offering sufficient and well-paid employment opportunities in the rural areas. This view is reflected by responses in our study, with 81.3% indicating that financial concerns were an impediment to working in the rural areas (Table 2). Similarly, 61% of the respondents indicated the potential to earn a good income as motivation to accepting a government job in the rural area and 88.8% indicated that this would be a very attractive incentive. These incentives referred to above, together with others discussed below in this article, suggest that less restrictive options (compared to the certificate of need) may attract more optometry graduates to the rural areas where their services are so desperately needed. If the rural community dwellers cannot afford the cost of eye care services, these services may need to be provided as part of public healthcare, with government needing to explore the possibility of providing more eye care services in rural areas.

Sacharowitz35 indicated that one of the greatest barriers to providing eye care services in South Africa, particularly in the rural areas, is that the previous health policies did not make provision for employing optometrists in government hospitals and clinics. However, Limpopo and KwaZulu-Natal Provinces are now prioritising eye care services as one of the healthcare focus areas, but it is taking a while for the other provinces to do the same. The establishment of posts and budgets for optometrists’ salaries in the government or public health sector seem relatively straightforward. However, the financing of the provision of ophthalmic devices would be a major obstacle. It is important to acknowledge the efforts of NGOs, which provide spectacles free of charge in many government or public health sector facilities. It is suggested that the government partner with these NGOs by providing subsidies for spectacles and other assistive visual devices. In addition to ophthalmology clinics, the provincial Departments of Health has optometric services in many provincial hospitals, where they engage mostly in refraction.^36^ These government initiatives should be expanded to other provinces. The government's efforts to improve optometric services in rural areas by providing optometry students scholarships annually to attract them to government services following completion of their studies should be expanded.^36^ Salary packages for rural optometrists should be adjusted to include incentives such as rural allowances, grants for housing and free transportation. These incentives would attract new optometric graduates to rural areas and would be in line with the WHO^27^ recommendation of increasing access to health workers in remote and rural areas.

A notable outcome from the responses from students of a rural background was the desire to serve community eye care needs and a return to their hometown. This reinforces the need for outreach programmes to recruit optometry students from rural areas, as these students enter their professions with an awareness of rural living and the eye care needs of their hometown.

Compulsory community service is an attempt by the government to address the shortage of healthcare professionals, particularly in rural and under-resourced areas.^29^ It has been implemented for medical doctors, dentists, pharmacists, physiotherapists, occupational and speech therapists, clinical psychologists, dieticians, radiographers and environmental health officers. However, almost two decades post-apartheid, optometry in South Africa still does not offer community service opportunities.^37^ More than two-thirds of the respondents (69.6%) were not in favour of compulsory community service for graduating optometrists, of whom an overwhelming majority were from urban areas. These findings are not surprising, as a previous study has shown that the initial announcement regarding implementing community service for medical doctors was received negatively by the affected students.^29^ However, subsequent findings indicated that the majority of those eligible for community service did take up their posts and reported a positive experience.^29^

In this study, the majority of those who agreed with compulsory community service were from the rural areas. This is aligned with a report which indicates that social and personal reasons (the desire to help the community and close proximity to home) influence students’ desire to return to and work in their hometown. Community service for graduating optometrists can therefore serve as a platform for urban students to experience the nature of rural clinical settings, lifestyles and sense of community. The WHO27 recommendations imply that compulsory community service can improve appreciation of rural services and availability of health workers to rural areas. It is, therefore, recommended that the government speed up the implementation of community service for optometrists. This will help to identify the potential barriers to working in, as well as factors that may impede eventual deployment and retention in, rural areas.^38^

Other than economic (financial) incentives alone, personal factors (favourable working environment and better living conditions) were rated by the majority of students influencing their decision not to work in a rural area (Table 3). Studies^28,29^ have shown that providing appropriate and adequate infrastructure, such as accommodation, is an important factor that would influence medical professionals to work in the rural areas. Although economic and personal factors were major considerations for students going to practise in the rural areas, it is also noteworthy that crime in the country was a major concern for the students (Table 2). Therefore, the issue of crime needs to be addressed by the government in order to attract and retain optometrists and other healthcare professionals in rural areas. Support and development (in terms of scholarship and bursary and career advancement) were also considered to be attractive options by both urban and rural students. Therefore, besides improving the supply of optometric manpower, training and career issues would also improve the standards of eye care services in the rural areas.

### Strengths and limitations of the study

The strength of this study is that it provides information that could improve the availability and accessibility of eye care services in the rural and remote areas of South Africa. This will help to reduce visual impairment in these parts of the country. A limitation of the study is that it did not assess the method of financing their education, which could have influenced the students’ responses. Another limitation is its quantitative nature and is therefore subjected to all the shortcomings of a quantitative study, such as limited in-depth understanding and investigation of the students’ responses.^39^

## Conclusion

This study has shown that few South African optometry students from urban areas have a desire to practise in rural areas after graduation, whilst those with a rural background are more inclined to pursue rural practice. However, many students, irrespective of location background, may opt to work in rural areas for NGOs or in the public sector after their training. Factors such as a conducive working environment, financial incentives, better living conditions and security featured as important factors that would attract graduating optometry students to rural areas, regardless of place of origin. The findings of this study have implications for the future of eye care services in the rural areas of South Africa. It is recommended that South African optometric institutions should accelerate and increase access and support for rural students with academic potential. There is also a need for establishing optometric training facilities in rural areas. It is acknowledged that implementing these recommendations may be challenging as they have serious financial implications for the government. A possible practical suggestion is that the government might have to look into strategies such as increasing health and education budgets in order to provide these needs.
